# The Contribution of Badgers to Confirmed Tuberculosis in Cattle in High-Incidence Areas in England

**DOI:** 10.1371/currents.outbreaks.097a904d3f3619db2fe78d24bc776098

**Published:** 2013-10-10

**Authors:** Christl A. Donnelly, Pierre Nouvellet

**Affiliations:** Imperial College London; Imperial College London

**Keywords:** badgers, cattle, culling, tuberculosis, vaccination

## Abstract

The role of badgers in the transmission and maintenance of bovine tuberculosis (TB) in British cattle is widely debated as part of the wider discussions on whether badger culling and/or badger vaccination should play a role in the government’s strategy to eradicate cattle TB. The key source of information on the contribution from badgers within high-cattle-TB-incidence areas of England is the Randomised Badger Culling Trial (RBCT), with two analyses providing estimates of the average overall contribution of badgers to confirmed cattle TB in these areas. A dynamical model characterizing the association between the estimated prevalence of Mycobacterium bovis (the causative agent of bovine TB) among badgers culled in the initial RBCT proactive culls and the incidence among sympatric cattle herds prior to culling is used to estimate the average overall contribution of badgers to confirmed TB herd breakdowns among proactively culled areas. The resulting estimate based on all data (52%) has considerable uncertainty (bootstrap 95% confidence interval (CI): 9.1-100%). Separate analyses of experimental data indicated that the largest estimated reduction in confirmed cattle TB achieved inside the proactive culling areas was 54% (overdispersion-adjusted 95% CI: 38-66%), providing a lower bound for the average overall contribution of badgers to confirmed cattle TB. Thus, taking into account both results, the best estimate of the average overall contribution of badgers is roughly half, with 38% being a robustly estimated lower bound. However, the dynamical model also suggested that only 5.7% (bootstrap 95% CI: 0.9-25%) of the transmission to cattle herds is badger-to-cattle with the remainder of the average overall contribution from badgers being in the form of onward cattle-to-cattle transmission. These estimates, confirming that badgers do play a role in bovine TB transmission, inform debate even if they do not point to a single way forward.

## Introduction

Wildlife reservoirs make the control of infectious diseases particularly challenging. Badgers (*Meles meles*) are a wildlife host for *Mycobacterium bovis*, the causative agent of tuberculosis (TB) in cattle^1^. Various badger-culling strategies have been used in Great Britain in attempts to reduce *M. bovis* transmission and to stop the spread of disease^1^. Annually repeated widespread ‘proactive’ culling, using cage-trapping and shooting, has been shown to reduce confirmed TB incidence in cattle herds within culled areas, but it has also been shown to increase risk among cattle herds on land outside, but within 2 km of, the culling area while culling was ongoing^2^^,^ 3 ^,^ 4 ^,^ 5 ^,^ 6. The disease system is complex, with transmission occurring both within and between species. Against this background policymakers and stakeholders have to weigh the potential benefits of particular disease control measures against their economic costs as well as other factors (including, for example, humaneness).


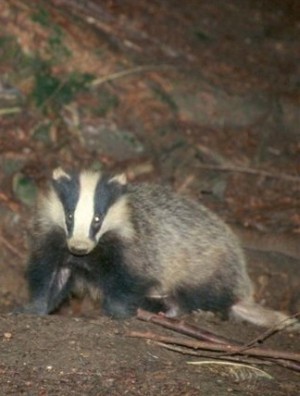


Badger (*Meles meles*) - Photo credited to Richard Yarnell.

Current approaches to limiting the contribution badgers make to cattle disease in Great Britain vary by region. In England, badger culling has been licensed; with two pilot culls currently underway^7^. These pilots are the first legal badger culls since 2005 (when the final proactive culls took place in the Randomised Badger Culling Trial, RBCT). In Wales, badgers are vaccinated (using an injectable vaccine) within a so-called Intensive Action Area^8^. Scotland is Officially TB Free and post-movement testing of cattle has been in place since 2005 to supplement statutory pre-movement TB testing and further reduce risks from the inward movement of cattle infected with *M. bovis* 9. Vaccine development is ongoing, with oral vaccination of badgers and injectable vaccination of cattle both being potential disease control options in the future.

Debate continues over whether or not any form of badger culling should be included in policies to control cattle TB in Great Britain. Although the debate is multi-faceted involving risk assessment, cost effectiveness, animal welfare, health and safety and ethics, much of the discussion has related to the potential level of benefits that might be achieved by a particular badger culling or vaccination policy. The RBCT provided clear evidence that annually repeated, widespread culling (termed 'proactive' culling) could significantly reduce confirmed cattle TB incidence within culled areas, while significantly increasing confirmed cattle TB incidence up to 2 km outside the culled areas while culling was ongoing^2^^,^ 3 ^,^ 4 ^,^ 5 ^,^ 6. The RBCT also provided evidence that a strategy of small localized culls (termed 'reactive' culling) could significantly increase cattle TB incidence across the 100 km^2^ areas randomized to this form of culling^10^^,^ 11.

The percentage of transmission to cattle herds, in a particular area, that is badger-to-cattle (whether transmitted directly or via bacterial contamination of the shared environment) determines the potential for badger-focussed control strategies to limit transmission to cattle but in a non-linear manner because substantial reductions in badger-to-cattle transmission over large areas would reduce onward transmission from cattle to other cattle (and thus from cattle back to badgers). Thus, the overall contribution of badgers to cattle TB is greater than the percentage of transmission to cattle herds that is badger-to-cattle.

Donnelly and Hone^12^ characterized the association between the estimated prevalence of *M. bovis* among badgers culled in the initial proactive culls in the RBCT and the incidence of confirmed TB among sympatric cattle herds prior to culling. The best-fitting model included frequency-dependent transmission between cattle herds with badger-to-herd transmission proportional to the estimated prevalence of *M. bovis* infection among the culled badgers. Inherent in this model was an estimate of the herd incidence level in the absence of transmission from badgers: 3.4% of herds (profile-likelihood-based 95% CI: 0 – 6.7%) would be expected to have TB infection newly detected per annum^12^, suggesting that herd-to-herd transmission was enough to sustain transmission in the absence of transmission from badgers. However, it was not possible to reject the null hypothesis that herd-to-herd transmission alone was insufficient to sustain bovine TB incidence in the cattle population (p=0.18)^12^.

Calculations subsequently undertaken based on the best-fitting model yielded an estimate (of roughly 50%) for the average overall contribution of badgers to confirmed cattle TB incidence in the RBCT areas (prior to culling)^13^. This estimate has informed discussions on limiting risks of badger transmission, but it lacked a confidence interval and sensitivity analysis. This paper provides further analysis of this estimate, its precision and its sensitivity.

Furthermore, this paper reviews the relevance of previously estimated reductions in confirmed cattle TB risk associated with proactive culling observed inside the RBCT culling areas and their implications for the average overall contribution of badgers to confirmed cattle TB.

## Methods

The RBCT quantified the impact of badger culling on TB incidence in cattle herds in areas of England where bovine TB is endemic. The RBCT compared the incidence of TB in cattle under three randomly allocated strategies – annually repeated widespread (‘proactive’) culling, localized (‘reactive’) culling around farms with confirmed TB breakdowns and no culling (‘survey-only’) – each replicated 10 times in 100 km^2^ trial areas recruited as matched sets of three areas, known as ‘triplets’^3^.

**Donnelly and Hone model**

The badger data analysed by Donnelly and Hone^12^ related to badgers culled in the initial proactive culls in the RBCT (excluding 19 badgers with incomplete data), as initially published by Woodroffe *et al*.^14^ (Table 1). The cattle herd incidence data analysed related to the 12-month periods preceding the initial proactive badger culls, as initially published by Donnelly *et al*.^2^ in the form of Supplementary Data based on location data as recorded in the VetNet database and reproduced by Donnelly and Hone^12^ (Table 1). It was critical that the cattle data related to a period of time before the initial proactive badger culls to avoid any impact of badger culling on the data analysed.

**Table 1:** Data on TB incidence among cattle herds in RBCT areas randomized to proactive badger culling (in the 12 months preceding the initial proactive cull) and on badgers culled in the initial proactive badger culls in the RBCT, as originally published by Woodroffe *et al*.^14^ and Donnelly *et al*.^2^ and analysed by Donnelly and Hone^12^. Note: a herd was said to experience a new 'confirmed' TB herd breakdown if one or more TB test-positive cattle (reactors) were identified while the herd was not under TB restrictions and post-mortem examination led to the detection of typical bovine TB lesions or culture of *M. bovis* in at least one reactor. ^1^ Based on the numbers of total herds and confirmed TB breakdown herds in the 12-month periods preceding the initial proactive badger culls, as initially published by Donnelly *et al*.^2^ in the form of Supplementary Data based on location data as recorded in the VetNet database. ^2^ Based on the numbers of badgers culled in initial proactive culls (excluding 19 with incomplete data), as initially published by Woodroffe *et al*.^14^ ^3^ Those badgers culled in initial proactive culls which tested positive for *M. bovis*, as initially published by Woodroffe *et al*.^14^

Triplet	Confirmed new herd breakdowns detected in the 12 months preceding the initial proactive cull ^1^	Total herds ^1^	Badgers culled ^2^	Infected badgers culled ^3^
A	8	71	55	8
B	15	152	238	13
C	8	105	243	4
D	11	97	293	102
E	4	116	602	29
F	4	138	446	13
G	7	245	422	29
H	11	63	161	12
I	15	100	218	82
J	8	114	442	65

The best-fitting model analysed by Donnelly and Hone^12^ included frequency-dependent between-herd transmission such that:

\begin{equation*}\frac{dU}{dt} = \frac{M}{p} - U \left( \beta \frac{I}{N} + \alpha \frac{I_B}{N_B} \right) \ \ \ \ \ \ \ \ \ \ \ \ \ {\rm Equation \ 1}\end{equation*}

\begin{equation*}\frac{dI}{dt} = U \left( \beta \frac{I}{N} + \alpha \frac{I_B}{N_B} \right) - Ic\end{equation*}

\begin{equation*}\frac{dM}{dt} = Ic - \frac{M}{p}\end{equation*}

where cattle herds transition between states *U* (uninfected), *I* (infected, and equivalently infectious, but undiagnosed) and *M* (under movement controls and thus assumed not infectious to other herds). *U*, *I* and *M* are the numbers of herds, rather than individual cattle, in each of these states and *N* is the total number of herds *(N=U+I+M).* Between-herd risk per annum is represented by β and the rate of infection from badgers per annum is represented by *α I_B_/N_B_* where *I_B_* denotes the number of infected badgers culled in the initial proactive cull in an RBCT area and *N_B_* denotes the total number of badgers culled in the initial proactive cull in an RBCT area. The per-annum rate at which infected herds are detected, and thus put under movement controls, is represented by *c*. The average time on movement control in years is represented by *p*. This model, like that of Barlow *et al*.^15^, assumed that any cattle-to-badger transmission events were negligible. This assumption is discussed in more detail below.

At equilibrium I* = M*/cp where I* and M* are at their equilibrium values. I* was obtained, as a function *I*_B_/*N*_B_, from the solution of the following quadratic equation (equation 16 of 12):

\begin{equation*}I*^{2} \left( \frac{\beta}{N} + p \frac{\beta}{N} c \right) + I* \left (- \beta + \alpha \frac{I_B}{N_B} + p c \alpha \frac{I_B}{N_B} + c \right) - N \alpha \frac{I_B}{N_B} = 0.\end{equation*}

As before, *p* was assumed to equal to 0.7 years (255 days) and

\begin{equation*}c = \frac{1}{0.86} \ \frac{2s}{b(s + 2(1-s))}  \approx 1.16 \ \frac{2s}{b(s + 2(1-s))} \end{equation*}

where *s* is the sensitivity of the herd test and *b* is the interval between routine herd tests (1 year for all herds in RBCT areas)^12^. The parameter *s* is assumed to equal 0.9 unless otherwise noted.

As in Donnelly and Hone^12^, estimates for α and β were obtained by maximum likelihood based on the log likelihood given by

\begin{equation*}l = B \ln \left( \frac{I*c}{N} \right) + (N-B) \ln \left( 1 - \frac{I*c}{N} \right)\end{equation*}

ignoring the additive constant where *B* is the number of herd breakdowns in a one-year period among *N* herds.

In this paper the confidence intervals for α and β were obtained by bootstrapping. This is in contrast to the earlier use of profile likelihoods to obtain confidence intervals^12^. A benefit of bootstrapping is that confidence intervals can be obtained easily for other quantities, including non-linear functions of α and β. This includes the proportion of herds expected to have TB infection newly detected in a year in the absence of transmission from badgers given by:

\begin{equation*}{\rm if} \ \ \beta>c, \  \pi(0) = c \left( 1 - \frac{c}{\beta} \right) \frac{1}{1+pc}; \ \ \ {\rm if} \ \ \beta \leq c, \ \pi(0) =0\end{equation*}

where *π*(*q*) denotes the proportion of herds expected to have TB infection newly detected in a year in areas in which *M. bovis* prevalence in badgers is *q*. The average overall contribution of badgers to confirmed cattle TB incidence in the RBCT areas (prior to culling) was thus obtained by averaging the triplet-specific estimates of this proportion:

\begin{equation*}\frac{ \pi \left( \frac{I_B}{N_B} \right) - \pi(0) }{\pi \left( \frac{I_B}{N_B} \right) }\ .\end{equation*}

The dynamical model also provides an expression for the percentage of transmission to cattle herds that is badger-to-cattle:

\begin{equation*}\frac{ \alpha \frac{I_B}{N_B}}{\beta \frac{I*}{N} + \alpha \frac{I_B}{N_B}}\end{equation*}

based on Equation 1 at equilibrium. The average percentage was similarly obtained by averaging the triplet-specific estimates of this percentage.

Mathematically there is no impact on any of the parameter estimates or maximum likelihood fit of incomplete diagnostic sensitivity in badgers (*s_B_*) of the standard necropsy undertaken in the RBCT^16^, except for a reinterpretation that α = α*/*s_B_*, assuming that *s_B _*was identical for in all triplets analysed.

For each estimate (including for (I*c)/*N*, the expected value of *B* given *I*_B_/*N*_B_), bootstrap 95% confidence intervals were obtained using two alternative methods. Non-parametric bootstrap samples were obtained by the sampling with replacement of the 10 RBCT areas. Parametric bootstrap samples were obtained with binomial resampling of the observed proportions (*B/N*) and (*I*_B_ */N*_B_) for each area. In each case 50,000 bootstrap samples were obtained for each assumed value of *s* (the sensitivity of the herd test). Non-parametric bootstrap confidence intervals, which are generally wider and therefore more conservative, are reported in the text; however, both types of bootstrap confidence intervals are reported in the tables to allow the reader to compare these alternative methods of quantifying parameter uncertainty. In addition, the estimated values associated with individual bootstrap samples can be plotted to show the bivariate distributions of two estimates or the univariate distributions of single estimates.

A potential criticism of the Donnelly and Hone^12^ model was that it made the simplifying assumption that cattle-to-badger transmission events were negligible within the context of the model. This may have been a particular issue for the analysis of data from the initial proactive culls undertaken in 2002 after the 2001 epidemic of foot-and-mouth disease (triplets D, I and J). It is known that the delayed testing and removal of TB-affected cattle caused by the FMD epidemic was associated with a significant rise in *M. bovis *prevalence in badgers^17^ as well as an increase in the incidence of confirmed TB breakdowns per herd. Thus, an alternative set of estimates was obtained excluding data from triplets D, I and J.

**Estimation of the impact of proactive culling on confirmed TB incidence in cattle**

Jenkins *et al*.^5^ used log-linear Poisson regression to compare the numbers of confirmed herd breakdowns recorded inside trial areas subjected to the proactive culling and inside trial areas with no culling (survey-only). The regression models adjusted for triplet, the log of the number of baseline herds at risk, and the log of the number of confirmed breakdowns recorded in a three-year period before RBCT culling. Confidence intervals were conservatively adjusted for extra-Poisson overdispersion by using an adjustment factor (the square root of the model deviance divided by the degrees of freedom) in all cases where its value was greater than 1.

Because proactive culling continued until 2005, the increased *M. bovis *prevalence among badgers observed in 2002^17^ is unlikely to have directly affected the estimated impact of proactive culling in the post-trial period (which began in 2006). Thus, the estimate presented is based on data from all 10 RBCT triplets.

## Results

**Donnelly and Hone model**

Assuming that diagnostic sensitivity of the herd test (*s*) was equal to 0.9, the maximum likelihood estimates of α and β were 0.047 (bootstrap 95% CI: 0.009 – 0.175) and 1.98 (bootstrap 95% CI: 1.57 – 2.12), respectively (Figures 1A, 2A, 2C, Table 2). Based on this model, 3.4% (bootstrap 95% CI: 0 – 8.5%) of herds would have been expected to have TB infection newly detected in a year in the absence of transmission from badgers. This corresponds to an average overall contribution of badgers to confirmed cattle TB of 52% (bootstrap 95% CI: 9.1%-100%) in the RBCT areas (prior to culling) (Figures 3A, 4A, 5A, 5C). The estimated average percentage of transmission to cattle herds that was badger-to-cattle was 5.7% (bootstrap 95% CI: 0.9-25%) (Figures 3C, 4C, 5A, 5C). These estimates and their precision varied little as a function of the assumed level of diagnostic sensitivity of the herd test (for *s*= 0.7, 0.8, 0.9 and 1; see Table 2). Furthermore, the maximized log likelihood varied little as a function of the assumed level of sensitivity.

**Table 2:** Estimates of α; β; the percentage of herds expected to have TB infection newly detected in a year in the absence of transmission from badgers (denoted π(0)); the average overall contribution of badgers to confirmed cattle TB incidence in the RBCT areas (prior to culling); and the average percentage of transmission to cattle herds that is badger-to-cattle with non-parametric [NP] and parametric [P] bootstrap 95% confidence intervals.

Dataset	Herd test sensitivity (*s*)	α	β	π(0)	Average overall contribution of badgers to confirmed cattle TB incidence	Average percentage of transmission to cattle herds that was badger-to-cattle	Maximized log likelihood
All 10 triplets	0.7	0.060 NP (0.012, 0.229) P (0.013, 0.168)	1.32 NP (0.97, 1.43) P (1.17, 1.39)	3.4% NP (0%, 8.5%) P (0%, 6.7%)	52% NP (9.2%, 100%) P (14%, 100%)	7.1% NP (1.2%, 31%) P (1.9%, 18%)	-316.76
All 10 triplets	0.8	0.053 NP (0.010, 0.199) P (0.011, 0.146)	1.62 NP (1.25, 1.75) P (1.46, 1.70)	3.4% NP (0%, 8.5%) P (0%, 6.7%)	52% NP (8.9%, 100%) P (14%, 100%)	6.3% NP (1.0%, 27%) P (1.6%, 16%)	-316.75
All 10 triplets	0.9	0.047 NP (0.009, 0.175) P (0.010, 0.129)	1.98 NP (1.57, 2.12) P (1.81, 2.07)	3.4% NP (0%, 8.5%) P (0%, 6.7%)	52% NP (9.1%, 100%) P (14%, 100%)	5.7% NP (0.9%, 25%) P (1.5%, 14%)	-316.74
All 10 triplets	1	0.043 NP (0.008, 0.155) P (0.009, 0.116)	2.42 NP (1.97, 2.57) P (2.22, 2.51)	3.5% NP (0%, 8.4%) P (0%, 6.8%)	52% NP (9.2%, 100%) P (14%, 100%)	5.2% NP (0.9%, 22%) P (1.3%, 13%)	-316.74
Omitting triplets D, I and J	0.9	0.045 NP (0.000, 1.69) P (0.000, 1.25)	1.99 NP (0.00, 2.07) P (0.00, 2.09)	3.6% NP (0%, 6.9%) P (0%, 7.3%)	42% NP (0.0%, 100%) P (0.0%, 100%)	3.7% NP (0.0%, 100%) P (0.0%, 100%)	-210.67

If data from triplets D, I and J were excluded from the analysis, the maximum likelihood estimates of α and β were 0.045 (bootstrap 95% CI: 0.000 – 1.69) and 1.98 (bootstrap 95% CI: 0.00 – 2.07), respectively (Figure 1B, 2B, 2D, Table 2). Based on this model, 3.6% (bootstrap 95% CI: 0 – 6.9%) of herds would have been expected to have TB infection newly detected in a year in the absence of transmission from badgers. This corresponds to an average overall contribution of badgers to confirmed cattle TB of 42% (bootstrap 95% CI: 0.0%-100%) in the RBCT areas (prior to culling) (Figures 3B, 4B, 5B, 5D). The estimated average percentage of transmission to cattle herds that was badger-to-cattle was 3.7% (bootstrap 95% CI: 0.0-100%) (Figures 3D, 4D, 5B, 5D).

**The proportion of herds in which confirmed cattle TB is newly detected within a year as a function of the observed prevalence of infection in badgers I_B/N_B.**
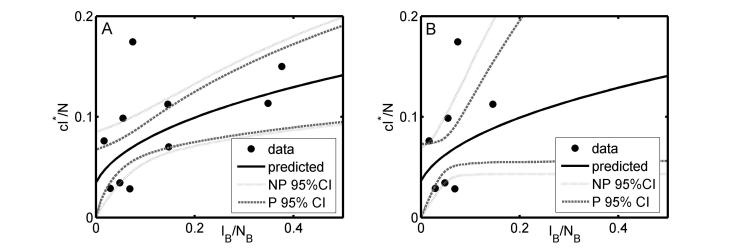
The black dots represent observed data: badger prevalence data are from badgers culled in the initial proactive culls in the RBCT and the cattle herd incidence data analysed relate to the 12-month periods preceding the initial proactive badger culls. The predicted line (I*c/N) was obtained assuming 90% herd test sensitivity for all ten RBCT triplets (panel A) and excluding triplets D, I and J (panel B). Pointwise 95% confidence limits were obtained using non-parametric (NP) and parametric (P) bootstrapping.

**The bivariate distributions of the estimates of alpha and beta (black dots) obtained from non-parametric (panels A and B) and parametric (panels C and D) bootstrap sampling.**
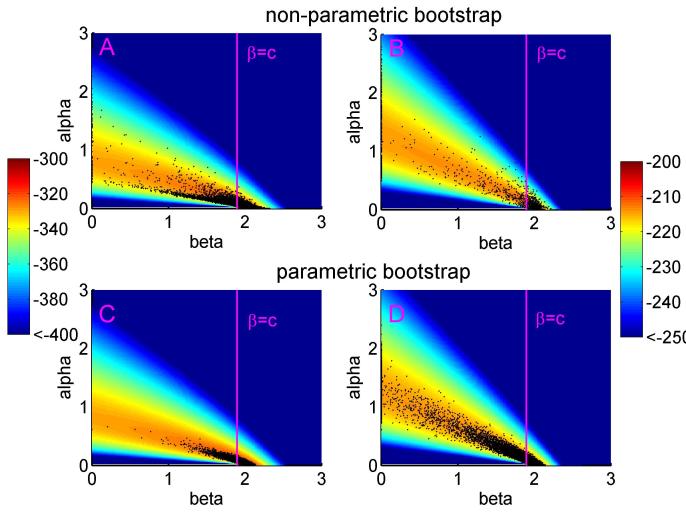
The background depicts the likelihood surface obtained from the original dataset, including all ten RBCT triplets (panels A and C) and excluding triplets D, I and J (panels B and D). The magenta vertical lines represent the critical values β = c. If estimates of β are less than or equal to *c*, then π(0)=0 and the overall contribution of badgers to confirmed cattle TB is 100%.

**The univariate distributions of the average overall contribution of badgers to confirmed cattle TB incidence (panels A and B) and the average percentage of transmission to cattle herds that was badger-to-cattle (panels C and D) obtained from non-parametric bootstrap sampling.**
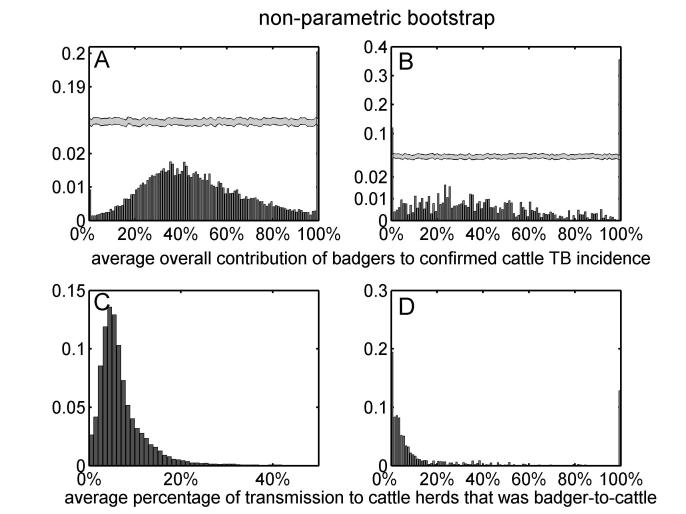
The univariate distributions of bootstrap estimates are based on all ten RBCT triplets (panels A and C) and excluding triplets D, I and J (panels B and D). Note that panels A and B have broken vertical axes.

**The univariate distributions of the average overall contribution of badgers to confirmed cattle TB incidence (panels A and B) and the average percentage of transmission to cattle herds that was badger-to-cattle (panels C and D) obtained from parametric bootstrap sampling.**
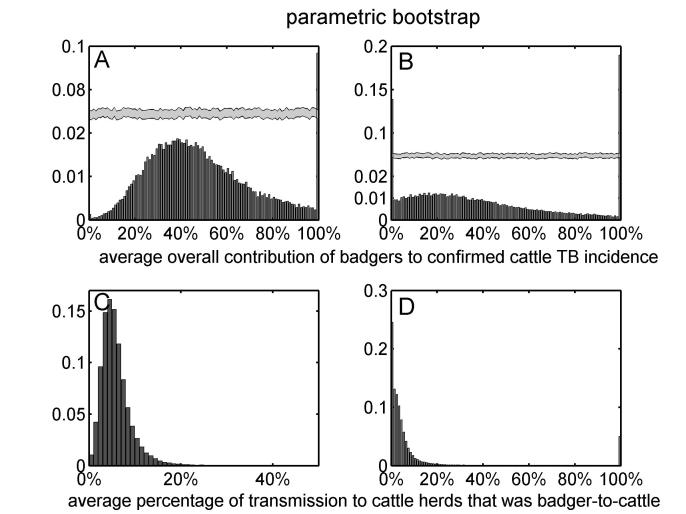
The univariate distributions of bootstrap estimates are based on all ten RBCT triplets (panels A and C) and excluding triplets D, I and J (panels B and D). Note that panels A and B have broken vertical axes.

**The bivariate distributions of the average overall contribution of badgers to confirmed cattle TB incidence and the average percentage of transmission to cattle herds that was badger-to-cattle.**
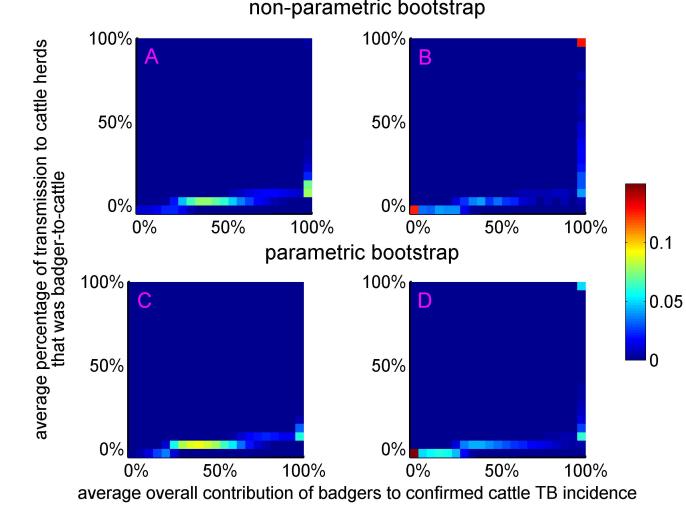
The colour scale corresponds to the proportion of bootstrap estimates that fell within the 5%-by-5% wide bins. The bivariate distributions were obtained from non-parametric (panels A and B) and parametric (panels C and D) bootstrap sampling, including all ten RBCT triplets (panels A and C) and excluding triplets D, I and J (panels B and D).

**Estimation of the impact of proactive culling on confirmed TB incidence in cattle**

The largest reduction in confirmed cattle TB incidence achieved over the course of the RBCT was that within RBCT trial areas during the first 18-month time period post-trial (that is between 12 and 30 months after the final annual proactive cull in each of the 10 proactive trial areas). The estimated reduction over this period was 54% with an overdispersion-adjusted 95% confidence interval of 38% to 66%^5^. Because badgers were never completely eliminated from any of the culling areas and farmers moved cattle in and out of the areas, the lower confidence bound of this estimate can provide a lower bound for the average overall contribution of badgers to confirmed cattle TB in the RBCT areas.

## Discussion

The confidence intervals obtained using bootstrap sampling were wider than the profile-likelihood-based intervals previously reported^12^. However, it was the exclusion of data from triplets D, I and J that considerably widened the confidence intervals obtained. Indeed, the confidence intervals obtained based on the restricted dataset for the average overall contribution of badgers to confirmed cattle TB incidence and the average percentage of transmission to cattle herds that was badger-to-cattle spanned from 0 to 100%.

Taking into account all of the available scientific evidence from all 10 triplets in the RBCT, the best estimate of the average overall contribution of badgers to confirmed cattle TB within high-cattle-TB-incidence areas is roughly half, with 38% being a robustly estimated lower bound and no robustly estimated upper bound.

Although an average overall contribution from badgers of at least 38% indicates their importance in the disease system (at least within the areas analysed), it does not indicate how this contribution might best be limited. Badger vaccination with injectable BCG (in Wales) and badger culling (England) are both currently underway as part of government policies. The former was adopted based on evidence that injectable badger vaccines reduce disease in badgers^18^, and the latter was adopted based on evidence from the RBCT that annually repeated widespread badger culls over large areas can yield net reductions in cattle herd incidence in the affected area (the culling area and up to 2 km outside of it)^2^^,^ 3 ^,^ 4 ^,^ 5 ^,^ 6. However, it should be noted that the controlled shooting of free-ranging badgers, allowed in the pilot badger culls currently underway in England, was not part of the RBCT (which relied on shorter bouts of cage trapping badgers before they were culled).

The 54% reduction (overdispersion-adjusted 95% confidence interval: 38% to 66%) in confirmed cattle TB incidence was only within RBCT trial areas for an 18-month time period between 12 and 30 months after the final annual proactive cull in each of the 10 proactive trial areas, following an average of 5 years of proactive culling. Long-term estimates (over 9.5 years including 5 years of culling) of the average net impact of proactive culling of circular areas of 150km^2^were 3–22% (central figure 12%) or 8–24% (central figure 16%), depending on particular assumptions^19^. At present there is no evidence for the impact of badger vaccination on TB incidence in cattle because no study to measure the impact has been conducted. However, there is evidence from a field trial that badger vaccination significantly reduced the risk of vaccinated badgers testing positive to the available live tests of infection^20^^,^ 21.

According to the analyses presented, although a complete absence of transmission from badgers from the system is predicted to halve the incidence of TB in cattle (with a 38% reduction being a robustly estimated lower bound), only 5.7% (bootstrap 95% confidence interval: 0.9%-25%) of transmissions to herds were estimated to have been badger to cattle. However, the data analysed related to initial proactive culls in the RBCT, which took place between 1998 and 2002. Since this time, statutory pre-movement TB testing of cattle was introduced to reduce risks from the inward movement of cattle infected with *M. bovis*. By reducing herd-to-herd transmission events, pre-movement testing would likely have increased both the overall contribution of badgers to confirmed cattle TB incidence and the percentage of transmission to cattle herds that was badger-to-cattle.

Clearly derived statistical estimates are very important in a debate where some stakeholders maintain that badgers do not transmit TB to cattle, while others claim that the majority of cattle infections are acquired directly from badgers. Our findings show that badgers play a major role in maintaining *M. bovis* infection in England’s high incidence areas, but also indicate that badger-to-cattle transmission events are amplified by onward transmission within local cattle populations. Hence, our findings inform debate even if they do not point to a single way forward.
