# Supplementary material for: The Contribution of Badgers to Confirmed Tuberculosis in Cattle in High-Incidence Areas in England
Source: PLoS Curr. 2013 Oct 10;5:ecurrents.outbreaks.097a904d3f3619db2fe78d24bc776098. [Version 1] doi: 10.1371/currents.outbreaks.097a904d3f3619db2fe78d24bc776098 (PMC3992815; doi:10.1371/currents.outbreaks.097a904d3f3619db2fe78d24bc776098)
Supplement: Related texts

# Reply

**Published:** 2013-10-16

**Authors:** PLOS Currents

<strong>Media Coverage of this Article</strong> The following articles represent some of the media coverage that has occurred for this paper: Publication: BBC News Title: "New data on badger TB spread released" http://www.bbc.co.uk/news/science-environment-24493225 Publication: The Guardian Title: "How many cattle herds get TB from badgers?" http://www.theguardian.com/environment/damian-carrington-blog/2013/oct/11/badger-cull-cattle-tb-vaccination Publication: The Guardian Title: "The badger cull – key science questions answered" http://www.theguardian.com/environment/2013/oct/11/badger-cull-key-science-questions-answered Publication: The Independent Title: "Badgers cause 52% of TB infections in cattle, study finds" http://www.independent.co.uk/news/uk/politics/badgers-cause-52-of-tb-infections-in-cattle-study-finds-8875310.html?origin=internalSearch Publicaton: StatsLife Title: "Badgers – Estimating their role in cattle TB transmission" http://www.statslife.org.uk/features/999-badgers-estimating-their-role-in-cattle-tb-transmission
